# Effect of Laser Beam Oscillation on Thermal Behavior and Residual Stress in Multilayer Cu Foil Stacks to a Cu Tab Joint: A Numerical Investigation

**DOI:** 10.3390/ma19204391

**Published:** 2026-10-19

**Authors:** Bumsu Go, Kyounghak Kim, Heeseon Bang

**Affiliations:** 1 Research Institute, Korea Welding and Joining Engineering Association, Jeonnam-Gwangju Special Metropolitan City 61012, Republic of Korea; rhqjatn8@naver.com; 2 Department of Welding and Joining Science Engineering, Chosun University, Jeonnam-Gwangju Special Metropolitan City 61452, Republic of Korea; banghs@chosun.ac.kr

**Keywords:** foil stacks to tab welding, laser beam welding, oscillation, finite element analysis (FEA), residual stress, plastic strain

## Abstract

Laser welding of multilayer copper foil stacks for lithium-ion battery tabs generates steep thermal gradients that can induce plastic deformation and residual stresses, potentially affecting joint integrity. This study numerically investigates the effect of laser beam oscillation on the thermo-mechanical behavior of a 60-layer Cu foil stack joined to a 0.3 mm Cu tab using a three-dimensional sequentially coupled finite element model. Line and wobble welding were compared using temperature-dependent material properties and a moving conical Gaussian heat source. Beam oscillation increased the predicted peak temperature from 2060 K to 2577 K while reducing the through-thickness temperature reduction from approximately 22.5% to 12.1%, indicating a more uniform thermal field. The dominant longitudinal residual stress decreased from approximately 225 MPa to 181 MPa, while the von Mises equivalent residual stress decreased from 199 MPa to 141 MPa, corresponding to a reduction of approximately 29%. These results demonstrate that beam oscillation redistributes thermal energy and mitigates localized residual-stress concentration, providing a numerical basis for optimizing laser welding of multilayer Cu battery-tab joints.

## 1. Introduction

With the accelerating transition toward renewable energy, the market for electrical energy storage systems (ESSs) is undergoing rapid expansion. Owing to their high energy and power densities, lithium-ion batteries (LIBs) have emerged as the preferred option across diverse sectors, solidifying their position as the dominant technology for power tools, portable devices and electric vehicles (EVs).

The lithium-ion battery cells are manufactured by welding multiple layers of electrode foils to a tab to create the cell lead. Although ultrasonic welding is the preferred process for this procedure [1,2], there is a growing need for laser welding to overcome existing technical limitations and streamline production by eliminating a processing step [3,4]. It utilizes high production, less thermal energy, high flexibility, and easy automation with non-contact process.

High-power lasers with high density conventionally employed in industry typically operate at infrared (IR) wavelengths (e.g., 1064 nm). However, in battery welding applications, IR lasers are hindered by severe drawbacks, including elevated spatter, porosity, and defect formation, which collectively contribute to substantial mechanical and electrical variability. To address these limitations, green light lasers (wavelength 532 nm) have been developed [5], exhibiting significantly higher energy absorption on highly reflective surfaces. The wavelength-dependent absorptivity of various metals is well known. Specifically, compared with IR wavelengths, absorptivity at the green wavelength is approximately 8, 2.5, 1.5, and 1.2 times higher for copper, aluminum, nickel, and steel, respectively [6]. This enhanced absorption reduces the requisite energy input for the weld, thereby mitigating spatter formation and suppressing void generation during keyhole welding operations. Consequently, this broadens the processing window and yields welds with superior mechanical strength and reduced electrical resistivity by optimizing the weldability of highly conductive and reflective materials.

Grabmann et al. investigated the influence of process parameters (the laser power, the welding speed, the pulse duration and the pulse frequency) on mechanical properties of joints welded with a green high-power disk laser in lap joint configuration of a 40-layer foil stack to a lead tab (Cu to Cu and Al to Al) [7]. They reported the mechanical strength of the copper welds was about five times as high as for the aluminum welds. The maximum mean strength was 514 N for copper and 92 N for the aluminum stack, as the mechanical strength of the aluminum joint was reduced by the occurrence of detachments of the weld seams from the foils. Rinker et al. investigated welding limits of multiple Cu foil stacks to a tab from 30 layers to 90 layers using green laser welding [3]. As the number of foil layers increased, the process window was reduced due to the increasing number of defects present.

On the other hand, laser oscillating welding utilizes the deflection capabilities of a galvanometer scanner to execute complex beam trajectories, thereby preserving the inherent advantages of conventional laser welding. Furthermore, the application of beam oscillation concurrently alleviates the strict assembly tolerances typically required by conventional laser welding processes [8]. A few studies reporting on overlap joint configuration using green lasers studied multilayer welding of electrode Cu (or Al) foil stacks to a tab. Hong et al. systematically investigated the joining characteristics of a 60-layer Cu foil stack to a Cu lead tab using green laser welding and examined the influence of lap configuration (upper tab and upper foil stacks) and seam strategies (line and wobble) [9]. The wobble-based seam strategy produced a wider effective bonded region and improved joint integrity in the foil-stack-top configuration, reaching a maximum tensile shear load with reduced electrical resistance.

Notably, in conventional non-oscillating laser welding, process parameters such as laser power and welding speed dictate the heat input per unit length. This, in turn, governs the peak temperature and cooling rate, ultimately determining the magnitude and distribution of residual stresses [10,11]. However, with the implementation of beam oscillation, oscillation amplitude and frequency emerge as critical parameters influencing residual stress evolution [12,13].

In summary, several studies reporting the effect of laser beam oscillation welding on weldability, including mechanical properties and metallurgical characteristics, were performed. However, with the exception of a few studies [14,15], there has rarely been a simulation approach investigated that could evaluate structural performance for residual stress in multiple Al or Cu foil stacks to tab welds. Therefore, the aim of this study is to investigate the evaluation of the structural integrity through prediction of welding residual stress in 60-layer Cu foil stacks to a 0.3 mm Cu tab joint using a three-dimensional transient thermal conduction analysis and thermo-elastic-plastic analysis.

## 2. Research Methods

### 2.1. Finite Element Modeling

**Figure 1:**
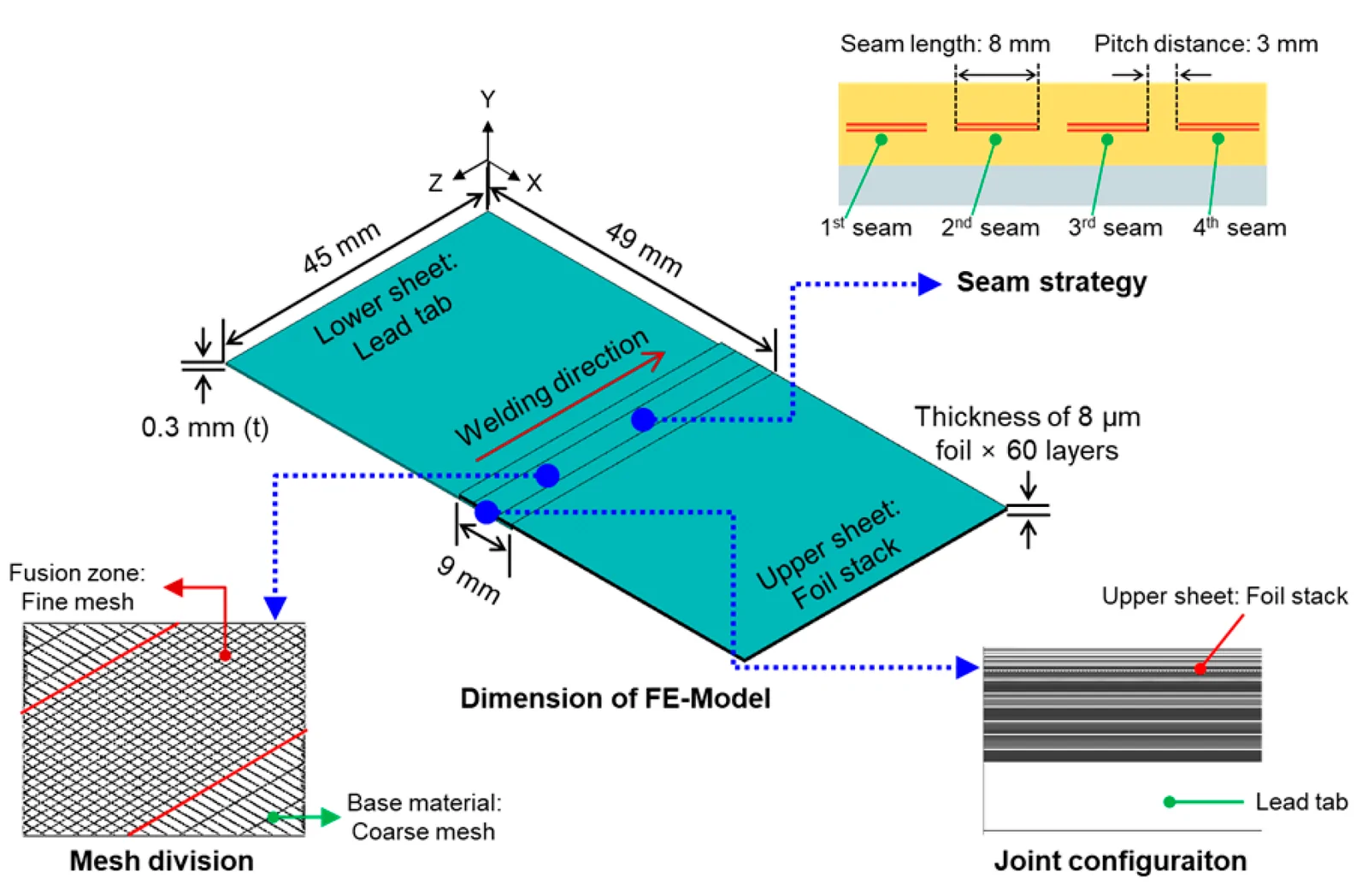
Finite element modeling of laser-welded joints.

Numerical simulation was conducted to evaluate the temperature distribution, welding residual stress, and plastic strain distribution of the joint between the foil stack and the lead tab using the commercial finite element (FE) software ANSYS 16.0. The dimensions of the simulated lead tab and foil stack were identical to those of the experimental specimens (shown in Figure 1), measuring 45 mm (L) × 49 mm (W) × 0.3 mm (t) and 45 mm (L) × 49 mm (W) × 0.8 µm (t) (comprising 60 layers), respectively, as shown in Figure 1. The FE model was meshed with SOLID70, an isoparametric 3D solid element suitable for steady-state and transient thermal analyses. This element features eight nodes, with temperature defined as a single degree of freedom at each node. For the subsequent thermo-elastic-plastic analysis, the SOLID185 element was employed, utilizing the thermal analysis results as thermal load input data. To accurately capture the large temperature gradients during the welding process, a highly refined mesh was applied along the weld line, which gradually coarsened toward the base material. The minimum element size was set to 0.2 mm × 0.2 mm × 0.008 mm. Ultimately, the constructed FE model comprised 138,467 nodes and 117,000 elements. In case of boundary conditions for the thermal conduction analysis, a convective heat transfer coefficient of 20 × 10^−6^ W/(mm^2^·K) was applied to all external surfaces. Both the initial temperature of the base material and the ambient temperature were set to 300 K to simulate room-temperature conditions. In the thermo-elastic-plastic analysis, boundary conditions were established to permit the free expansion and contraction of the welded joint during the heat input and convective cooling phases. The numerical simulations evaluated two welding conditions for the foil stack and lead tab: conventional line welding for Case 1 and wobble welding for Case 2, as listed in Table 1. Based on our prior experimental investigation [9], representative laser welding conditions at 2000 W were chosen for the FEM analysis: 150 mm/s for line welding (from a tested range of 100–200 mm/s) and 50 mm/s with a 0.8 mm oscillation amplitude for wobble welding (from 0.4–1.2 mm). These parameters were selected via comprehensive evaluations of bead morphology, mechanical properties, and electrical resistance.

### 2.2. Numerical Simulation

#### 2.2.1. Thermal Analysis

The three-dimensional transient heat conduction analysis of laser welding must account for the moving heat source, as the position of the laser beam changes over time. Additionally, the rapid temperature changes during the welding process necessitate consideration of the temperature-dependent physical properties, such as thermal conductivity, density and specific heat. These factors are essential for achieving accurate heat conduction analysis.

The governing equation of three-dimensional transient heat conduction analysis in joining of a foil stack to a tab by the laser welding process is defined as follows:

(1) $$\rho c\frac{\partial T}{\partial t}=\lambda \left(\frac{\partial^{2}T}{\partial x^{2}}+\frac{\partial^{2}T}{\partial y^{2}}+\frac{\partial^{2}T}{\partial z^{2}}\right)+\dot{Q}$$

where λ is the thermal conductivity of isotropic material (W/mm·K), T is the temperature (K), ρ is the density (kg/mm^3^), $\dot{Q}$ is the internal heat generation per unit volume (W/mm^3^), t is the time (sec), and c is the specific heat (J/kg·K).

In terms of boundary conditions, the convective heat transfer with the ambient environment based on Newton’s law was considered on all external surfaces, as follows:

(2) $$q_{c}=h_{c}(T_{ext}-T)$$

where $q_{c}$ is the heat dissipation, $h_{c}$ is the heat transfer coefficient (W/ mm^2^·K), $T_{ext}$ is the geometry temperature (K), and T is the ambient temperature (K).

#### 2.2.2. Mechanical Analysis

The temperature history calculated in the temperature field model was applied as a thermal load for elastic-plastic analysis of the residual stress field. The elastic strain–stress relationship in the elastic region is considered by isotropic Hooke’s law. The plastic behavior in the plastic region is considered by the von Mises yield criterion and linear isotropic hardening rule.

The total strain increment at any material point was decomposed into elastic, plastic, and thermal components as follows:

(3) $${\dot{\varepsilon }}_{ij}^{total}={\dot{\varepsilon }}_{ij}^{e}+{\dot{\varepsilon }}_{ij}^{p}+{\dot{\varepsilon }}_{ij}^{th}$$

where ${\dot{\varepsilon }}_{ij}^{total}$ is the total strain rate, ${\dot{\varepsilon }}_{ij}^{e}$ is the elastic strain rate, ${\dot{\varepsilon }}_{ij}^{p}$ is the plastic strain rate, and ${\dot{\varepsilon }}_{ij}^{th}$ is the thermal strain rate.

The thermal strain rate, which is calculated by the amount of change in temperature multiplied by the thermal expansion coefficient, can be expressed as follows:

(4) $${\dot{\varepsilon }}_{ij}^{th}=\delta_{ij}\alpha \dot{T}$$

where $\delta_{ij}$ is the Kronecker delta function, α is the thermal expansion coefficient, and $\dot{T}$ is the rate of temperature change.

By combining the strain rate components elastic, plastic and thermal, the total strain rate can be expressed as follows:

(5) $${\dot{\varepsilon }}_{ij}^{total}=\frac{1+v}{E}{\dot{\sigma }}_{ij}-\delta_{ij}\frac{v}{E}{\dot{\sigma }}_{kk}+\lambda s_{ij}+\delta_{ij}\alpha \dot{T}$$

where E is the Young’s modulus, v is Poisson’s ratio, λ is the plastic flow factor, $s_{ij}$ is the components of deviatoric stresses, and α is the thermal expansion coefficient. λ=0 is for elastic deformation ($\sigma_{e}<\sigma_{s}$), and λ>0 is for plastic deformation ($\sigma_{e}\geq \sigma_{s}$).

#### 2.2.3. Heat Source Model for Laser Welding

**Figure 2:**
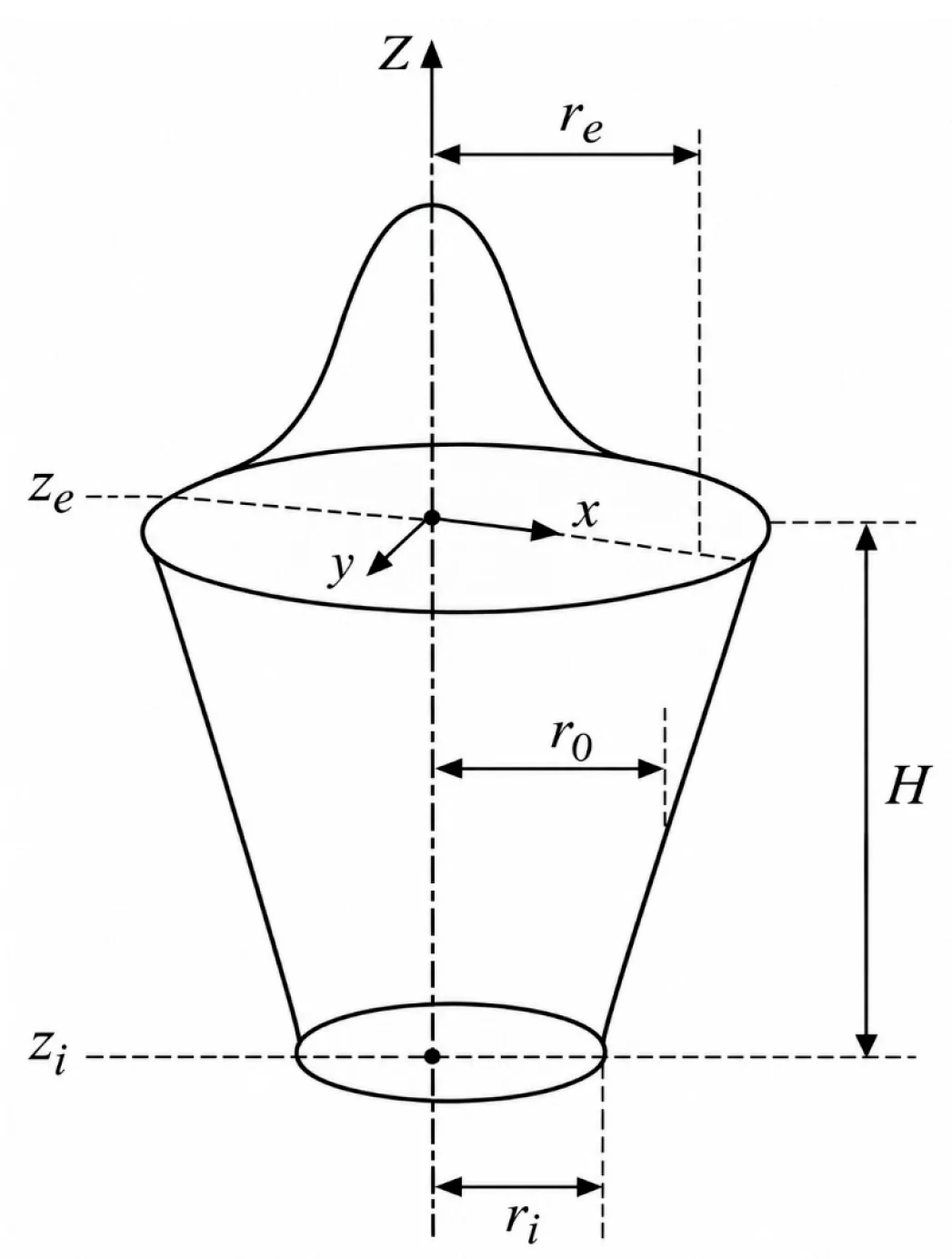
Conical Gaussian volumetric heat input model [12].

As shown in Figure 2, a three-dimensional conical Gaussian heat source was adopted to represent the laser heat input. Its heat flux distribution is expressed as follows [12]:

(6) $$q(r,\;z)=\frac{9\eta Qe^{3}}{\pi H(e^{3}-1)(r_{e}^{2}+r_{e}r_{i}+r_{i}^{2})}exp\left(-\frac{{3r}^{2}}{r_{0}^{2}}\right)$$

(7) $$r_{0}=r_{e}-\frac{\left(r_{e}-r_{i})(z_{e}-z\right)}{z_{e}-z_{i}}$$

(8) $$H=z_{e}-z_{i}$$

where $r_{e}$ is the radius of the upper surface, $r_{i}$ is the radius of the bottom surface, $z_{e}$ and $z_{i}$ are Z coordinates of the upper and bottom surfaces, respectively, Q is the laser welding power, and η is the laser energy absorption efficiency, which was set to 0.18 in the present study.

**Figure 3:**
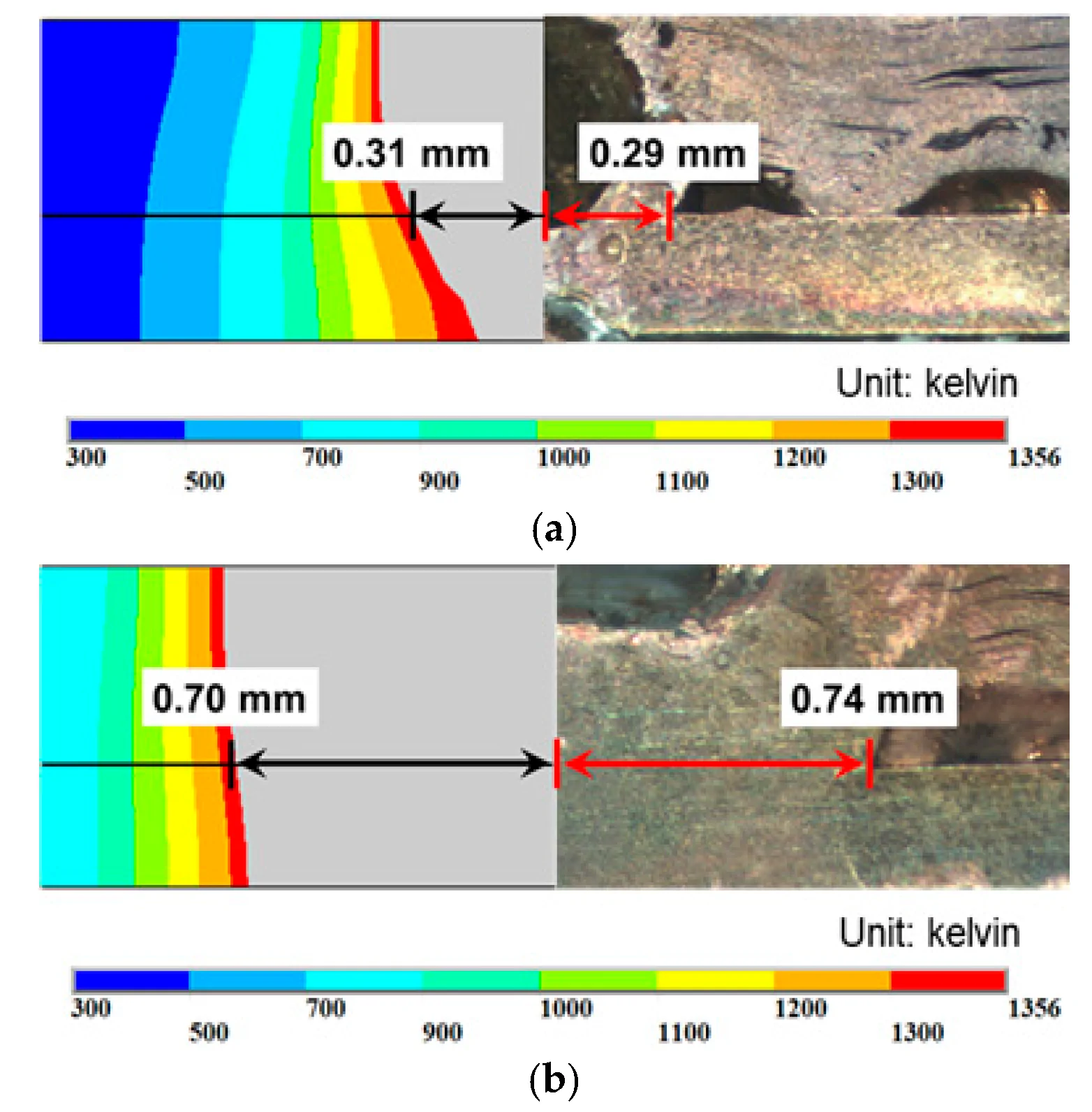
Comparison of the fusion zone shape between experimental and numerical analyses of line- and wobble-welded joints. (**a**) Line welding; (**b**) wobble welding.

Figure 3 shows a comparison of the cross-sectional view at the center of the weld line between the line- and wobble-welded joints obtained from the numerical analysis and the experiment; the gray region indicates the fusion zone, where the melting temperature of copper exceeds 1356 K. The laser beam is concentrated on the weld seam, resulting in a narrow fusion zone and a relatively small interface width of the welded joint (Figure 3a). In contrast, as shown in Figure 3b, when beam oscillation is applied, the beam energy is widely distributed along the oscillating laser trajectory, leading to a more uniform fusion zone and a wider interface width of the welded joint. Generally, the boundaries of the fusion zones for the line- and wobble-welded joints obtained numerically were similar in shape to those obtained experimentally. Therefore, it was confirmed that the welding heat source applied to numerical analysis of the FE model has good agreement with those obtained experimentally.

In three-dimensional transient heat conduction analysis, the position of the welding heat source varies with welding time, and it is necessary to account for the effects of heat source movement. This study aims to investigate the thermal and mechanical behavior of the welded joint with and without the application of oscillation. The moving heat source in line welding is as follows:

(9) $$\left(x_{\left(t\right)},y_{\left(t\right)})=(x_{0}+vt,\;y_{0}\right)$$

where v is the welding speed, and t is the welding time.

**Figure 4:**
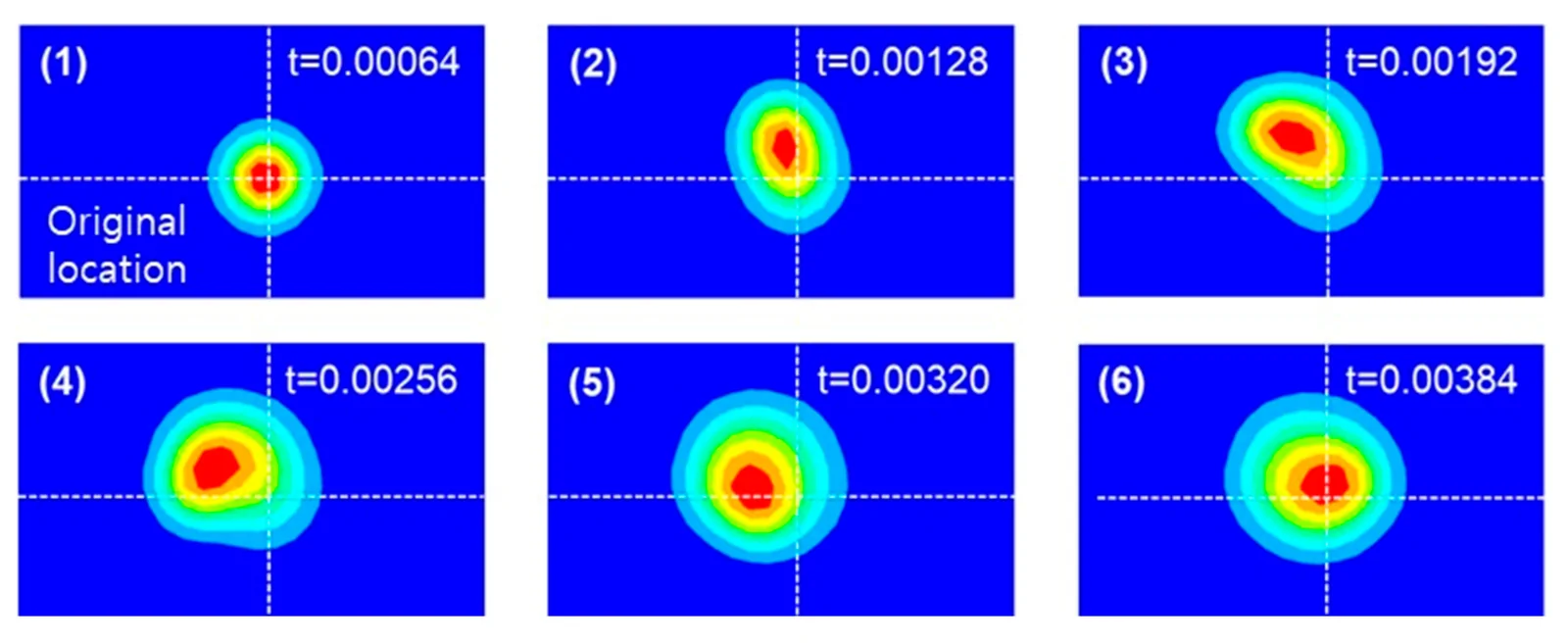
Oscillation cycle of wobble welding.

As shown in Figure 4, the moving heat source with oscillation, where the position of the laser beam oscillates periodically with welding time, wobble amplitude, and oscillation frequency as a combination of sine and cosine functions in the x–y plane, can be modeled and expressed as follows [16,17]:

(10) $$\left(x_{\left(t\right)},y_{\left(t\right)}\right)=[x_{0}+vt-A\cos(2\pi ft),\;y_{0}+A\sin\left(2\pi ft\right)]$$

where A is the wobble amplitude, and f is the oscillation frequency.

### 2.3. Physical Properties and Mechanical Properties

**Figure 5:**
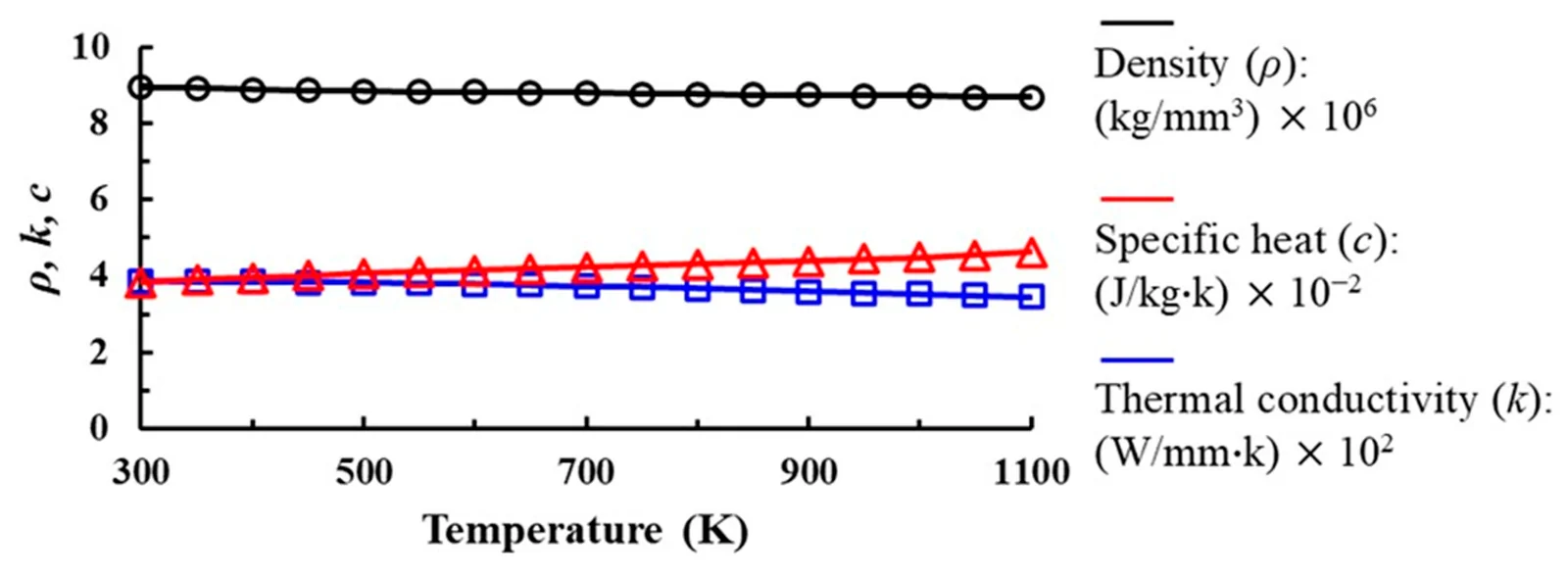

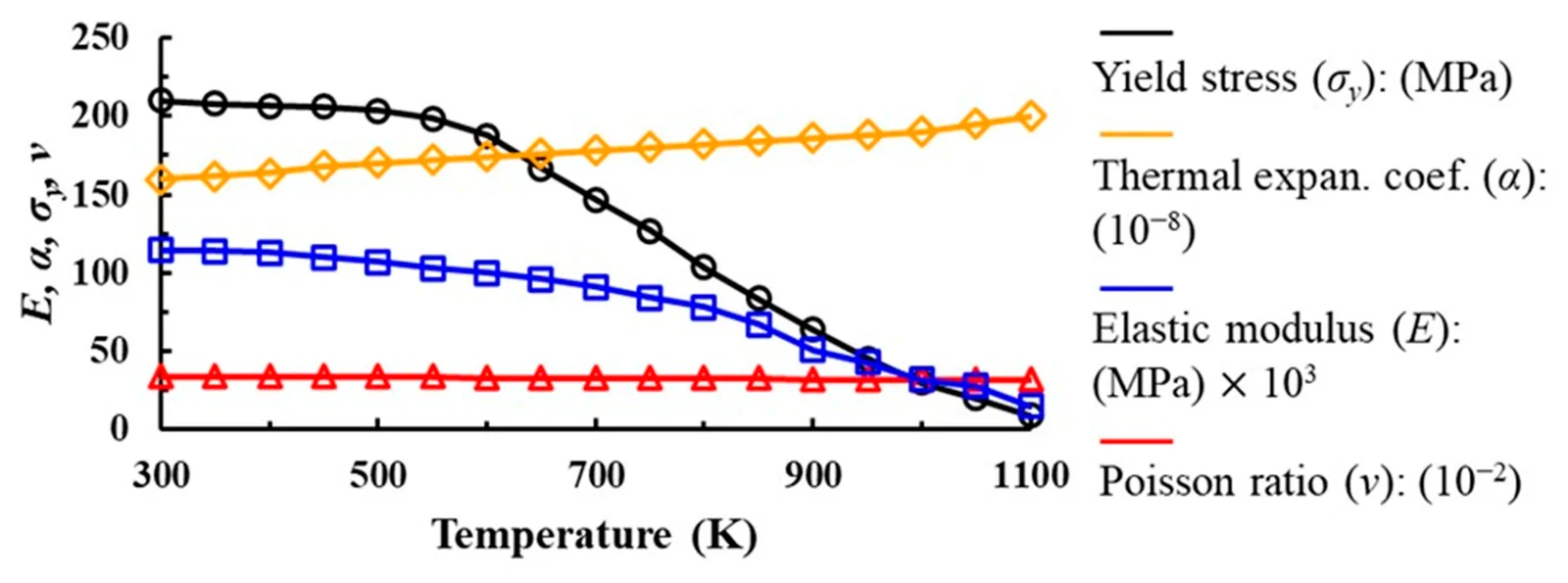
Thermal and mechanical properties of Cu [18,19].

**Figure 6:**
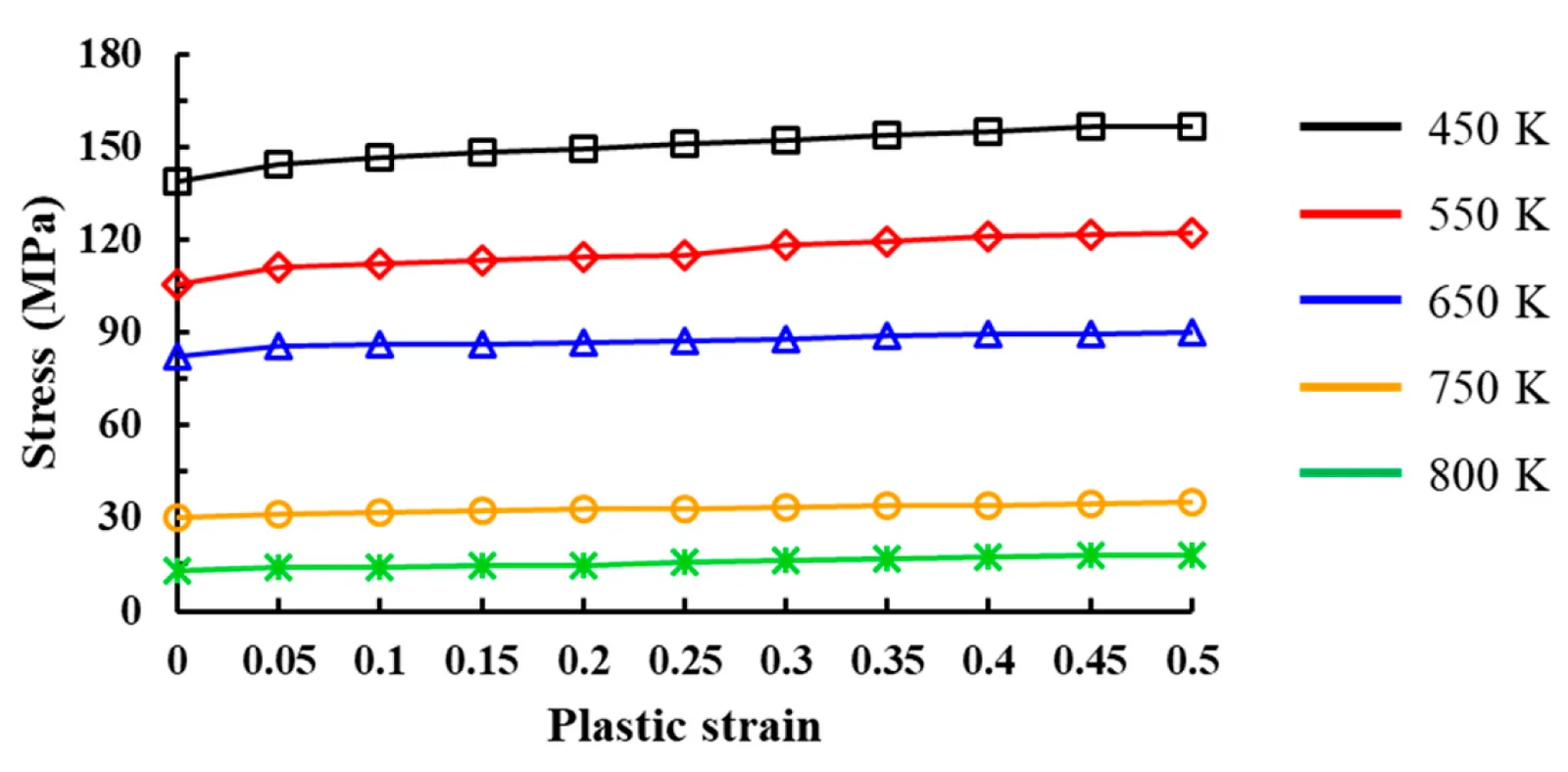
Stress–strain curve for Cu [20,21].

The temperature-dependent thermal physical properties and mechanical properties of pure copper applied in the present analysis are shown in Figure 5. The temperature-dependent thermal physical properties such as density, specific heat and thermal conductivity are used for transient thermal analysis, and the temperature-dependent mechanical properties, including elastic modulus, Poisson ratio, yield stress and thermal expansion coefficient, are used for thermo-elastic-plastic analysis [18,19]. Furthermore, as shown in Figure 6, the plastic behavior in the plastic region is considered using the von Mises yield criterion and the linear isotropic hardening rule with a bilinear tangent modulus [20,21].

## 3. Results and Discussion

### 3.1. Temperature Field

**Figure 7:**
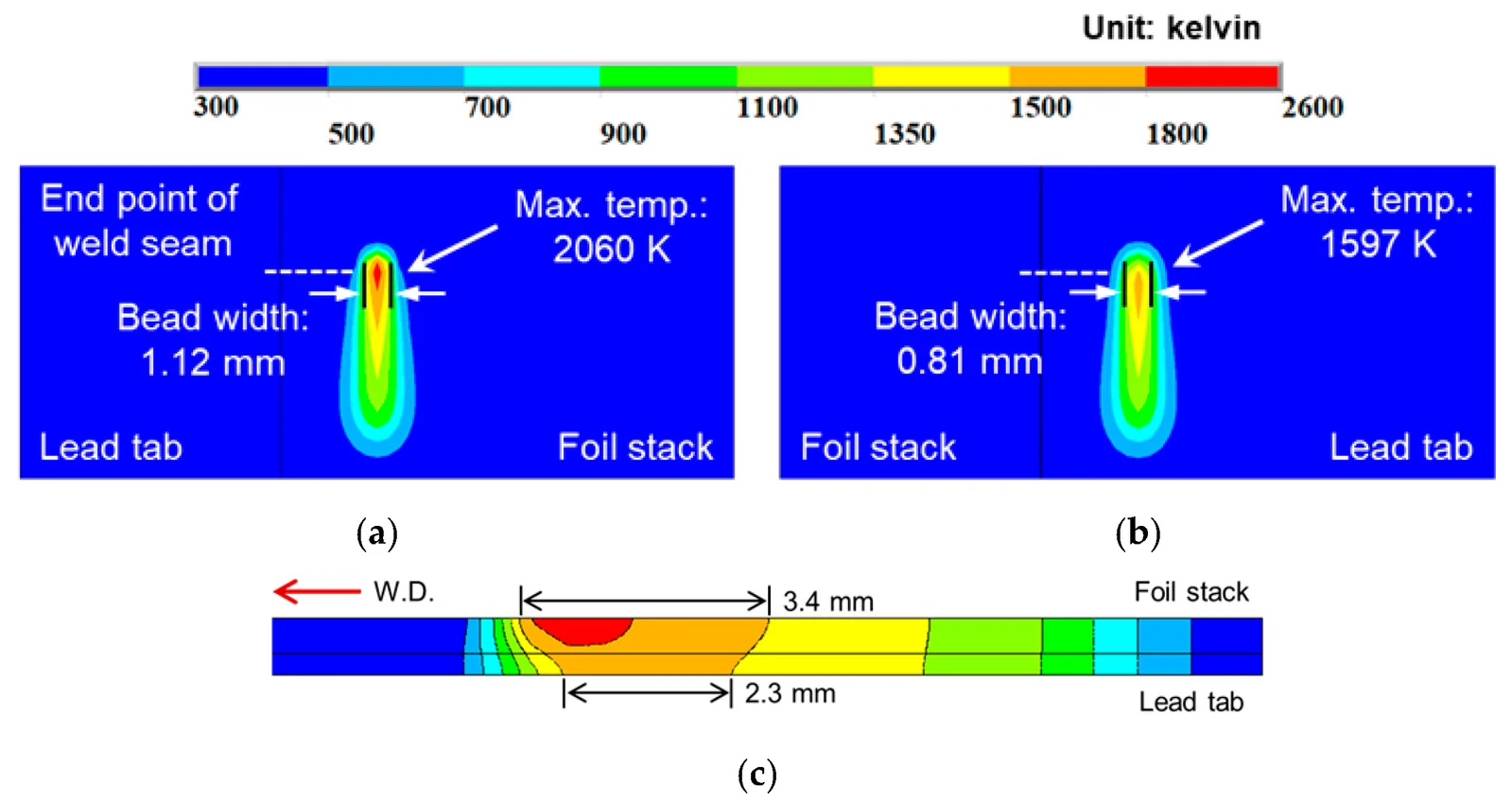
Temperature distribution in line welding (without oscillation). (**a**) Top surface, (**b**) bottom surface and (**c**) longitudinal cross section.

Figure 7 presents temperature distribution at the end point of the first stitch seam in line welding (without oscillation) of multiple Cu foil stack to tab joints. As shown in Figure 7a and Figure 7b, the maximum temperatures of the fusion zone at the top and bottom surface were 2060 K and 1597 K, respectively, while the bead widths were 1.12 mm and 0.81 mm, respectively. It can be inferred that the thermal diffusion within the material and heat loss to the surroundings reduce the amount of energy reaching the bottom surface as the laser beam energy transfers downward from the top surface. Consequently, the bottom surface exhibits a relatively lower maximum temperature and narrower bead width compared with the top surface. Moreover, as shown in Figure 7c, the temperature distribution in the longitudinal cross section reveals that the length of the fusion zone in the 1500 K range above the melting point was 3.4 mm and 2.3 mm on the top and bottom sides, respectively. As a result, an uneven thermal gradient along the weld line can be observed in line-welded joints. This is because the laser beam is concentrated on the weld line, transmitting high energy locally and causing a rapid temperature change.

**Figure 8:**
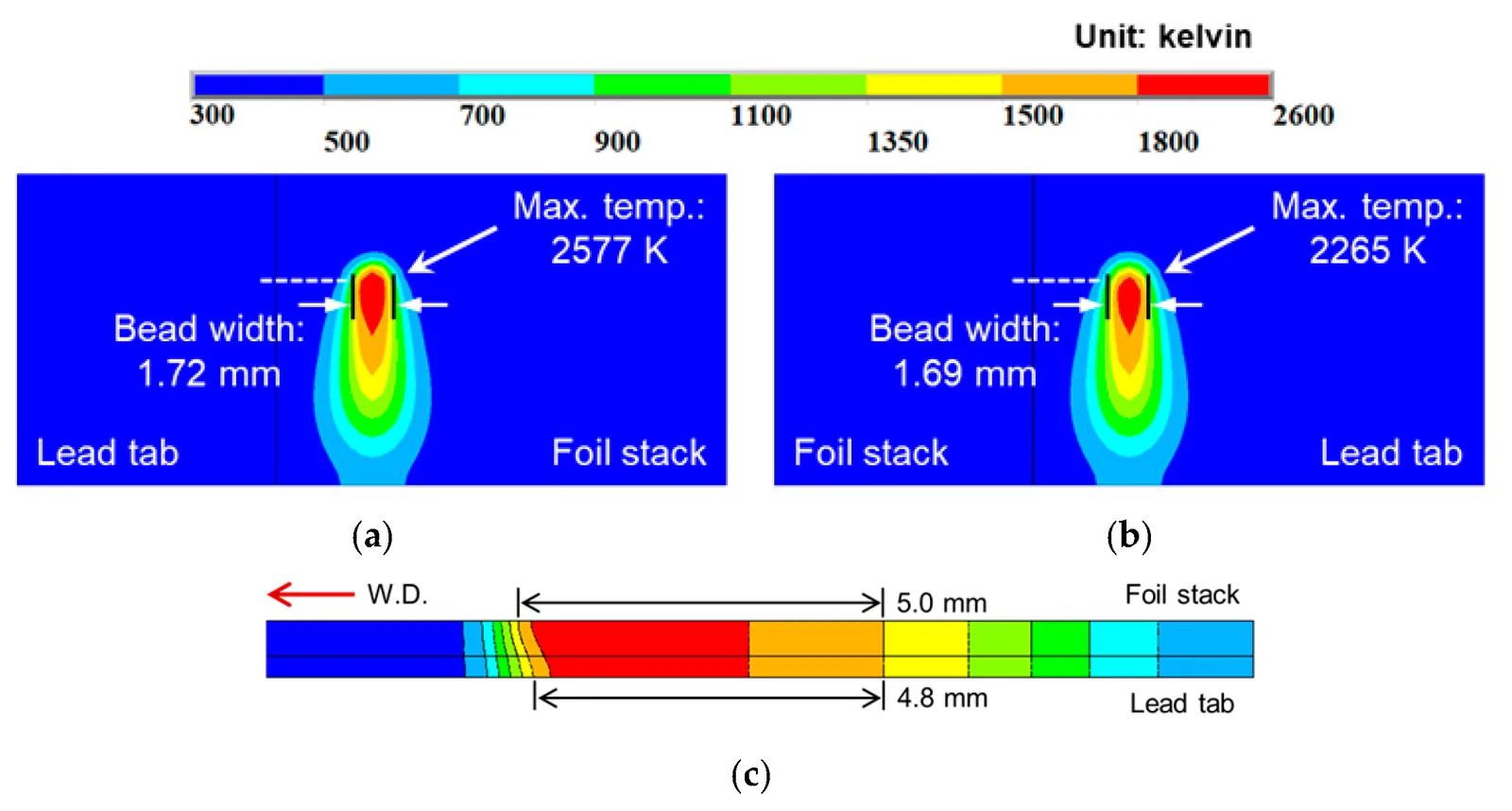
Temperature distribution in wobble welding (with oscillation). (**a**) Top surface, (**b**) bottom surface and (**c**) longitudinal cross section.

Figure 8 shows temperature distribution in wobble welding (with oscillation). The maximum temperatures of the fusion zone at the top and bottom surfaces were 2577 K and 2265 K, respectively, while the bead widths were 1.72 mm and 1.69 mm, respectively (Figure 8a,b). It can be observed that the maximum temperature of the molten zone and the bead width increase by 25% and 54%, respectively, compared with line welding. Additionally, the rear side of the fusion zone appears more rounded in shape compared with line welding, which indicates a more gradual thermal gradient in the width direction. Furthermore, as shown in Figure 8c, the temperature distribution in the 1500 K range above the melting point was 5.0 mm and 4.8 mm on the top and bottom sides, respectively. This indicates a more gradual thermal gradient along the weld line due to the application of beam oscillation. The differences in thermal behavior between line and wobble welding can be further explained by the combined effects of welding time, laser power, and beam oscillation kinematics. Although the laser power was maintained at 2000 W for both welding strategies, the lower welding speed employed in wobble welding (50 mm/s) compared with line welding (150 mm/s) resulted in a total welding time that was three times that of line welding for an equivalent weld length. Furthermore, the oscillatory beam motion at a frequency of 313 Hz and an amplitude of 0.8 mm modified the spatial and temporal distribution of the heat input, allowing repeated thermal exposure of regions along the welding path. These combined effects contributed to the higher predicted peak temperature and the broader high-temperature region observed in wobble welding. Therefore, the differences in the thermal behavior of the two welding strategies can be attributed to both the welding speed and the oscillatory motion of the laser beam rather than to beam oscillation alone.

**Figure 9:**
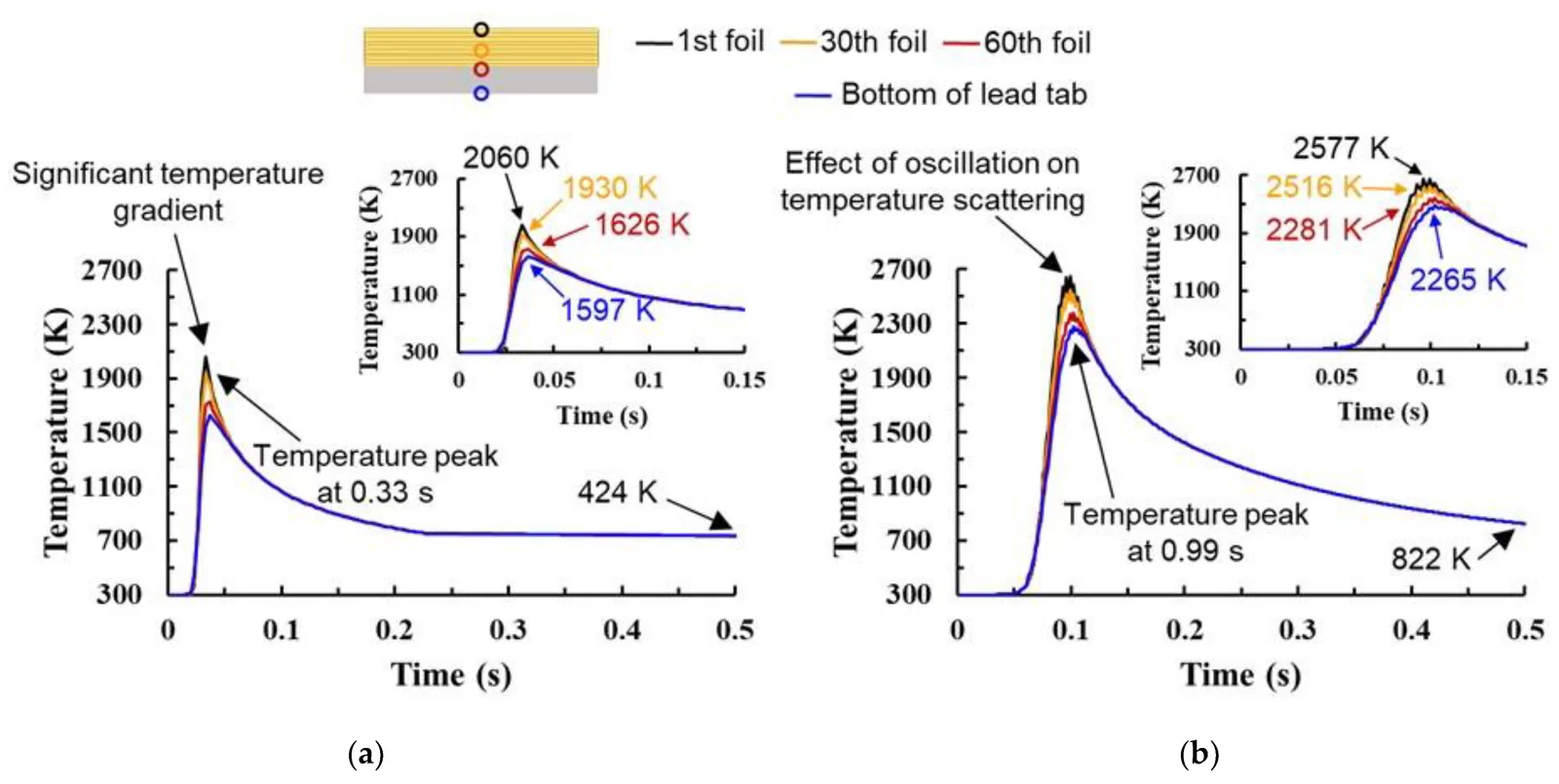
Temperature history of welded joints welding. (**a**) Line welding and (**b**) wobble welding.

In order to quantitatively examine the temperature reduction effect in the thickness direction of the foil stack to lead tab welded joint, the temperature histories at the first, 30th, and 60th foils and the tab bottom on the first stitch seam welding were investigated. Figure 9 presents the comparison of temperature history between line welding and wobble welding. As shown in Figure 9a, the maximum temperatures at four different points from the first foil to the bottom of the lead tab were 2060, 1930, 1626, and 1597 K, respectively. It can be observed that the temperature reduction rate from the first foil to the bottom of the lead tab was approximately 22.5%. This temperature reduction effect is supposed to potentially cause unstable weld quality as the number of foils increases going forward. Additionally, at the welding time of 0.033 s, when the maximum temperature is reached, the weld joint experiences faster heating and cooling rates in line welding. It can be inferred that this rapid temperature change leads to a significant thermal gradient. The temperatures at four different points were almost identical at 1210 K at a welding time of 0.077 s during the convection cooling stage, and then decreased to 424 K with a consistent temperature gradient after a welding time of 0.5 s. Figure 9b shows that the maximum temperatures at four different points from the first foil to the bottom of the lead tab were 2577, 2516, 2281, and 2265 K, respectively. The temperature reduction rate from the first foil to the bottom of the lead tab was approximately 12.1%. In contrast to line welding, which concentrates thermal energy locally, more uniform temperature distribution along the thickness direction is achieved in the wobble-welded joint due to the even distribution of thermal energy induced by beam oscillation. Additionally, at the welding time of 0.99 s, which corresponds to the peak temperature, a relatively gradual temperature change is observed in wobble welding. Especially, it can be clearly seen that the temperature scattering is caused by the circular motion resulting from the effect of beam oscillation. This suggests that a more gradual thermal gradient is achieved compared with line welding. The temperatures at four different points were almost identical at 1775 K at a welding time of 0.145 s during the convection cooling stage, and then decreased to 822 K after a welding time of 0.5 s.

### 3.2. Residual Stress and Plastic Strain Distribution

#### 3.2.1. Residual Stress Characteristics

**Figure 10:**
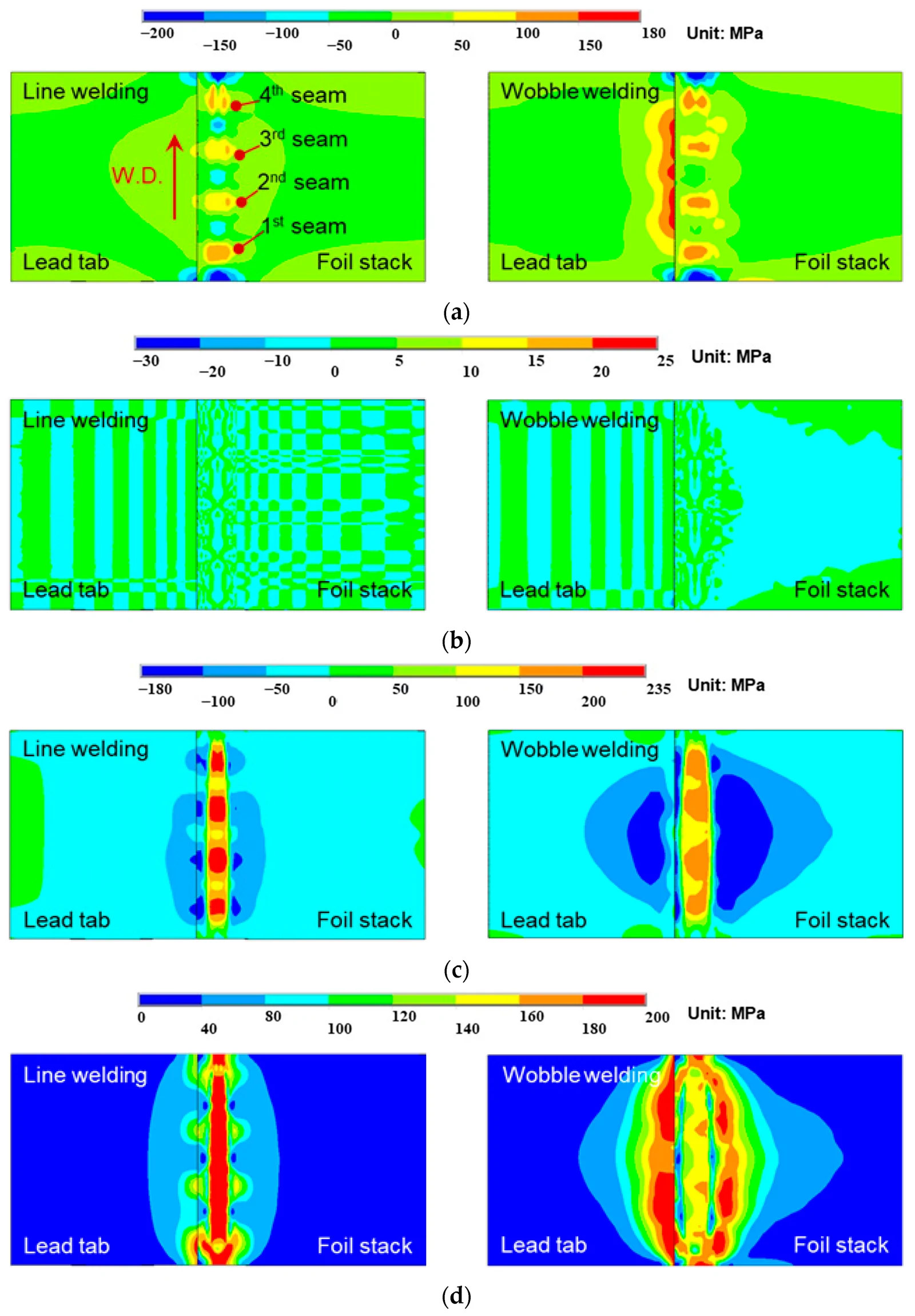
Comparison of residual stress distribution between line- and wobble-welded joints. (**a**) σxx (width direction), (**b**) σyy (thickness direction), (**c**) σzz (longitudinal direction) and (**d**) von Mises equivalent stress.

Figure 10 shows the comparison of welding residual stress distribution by stress components in different directions for line- and wobble-welded joints. In the case of residual stress in the width direction (Figure 10a), both line and wobble joints indicate tensile stresses of around 120 MPa in the region corresponding to the stitch seam. However, it can be observed that the distribution range of tensile stresses, including both foil stack and lead tab, is wider in the wobble welding compared with the line welding. This can be attributed to the increase in the molten pool area due to beam oscillation, as previously observed in Figure 8. It can be seen from Figure 10b that the residual stress in the thickness direction is approximately 0 MPa, which can be attributed to the minimized mechanical constraint effect in the thickness direction due to the dimensional characteristics of the ultra-thin material. Figure 10c shows the longitudinal component of residual stress distribution. It can be observed that high levels of tensile stresses (around 225 MPa) are concentrated at the stitch seam, slightly exceeding the yield stress of copper (210 MPa). In contrast, a reduced tensile stress of around 180 MPa is observed in the wobble-welded joint. These results indicate that the relatively uniform expansion and contraction in the fusion zone is caused by the uniform thermal gradient along the weld line direction due to beam oscillation. High levels of von Mises stress are concentrated at the weld line in the line-welded joint. In contrast, the stress is distributed around the weld line in the wobble-welded joint (Figure 10d).

**Figure 11:**
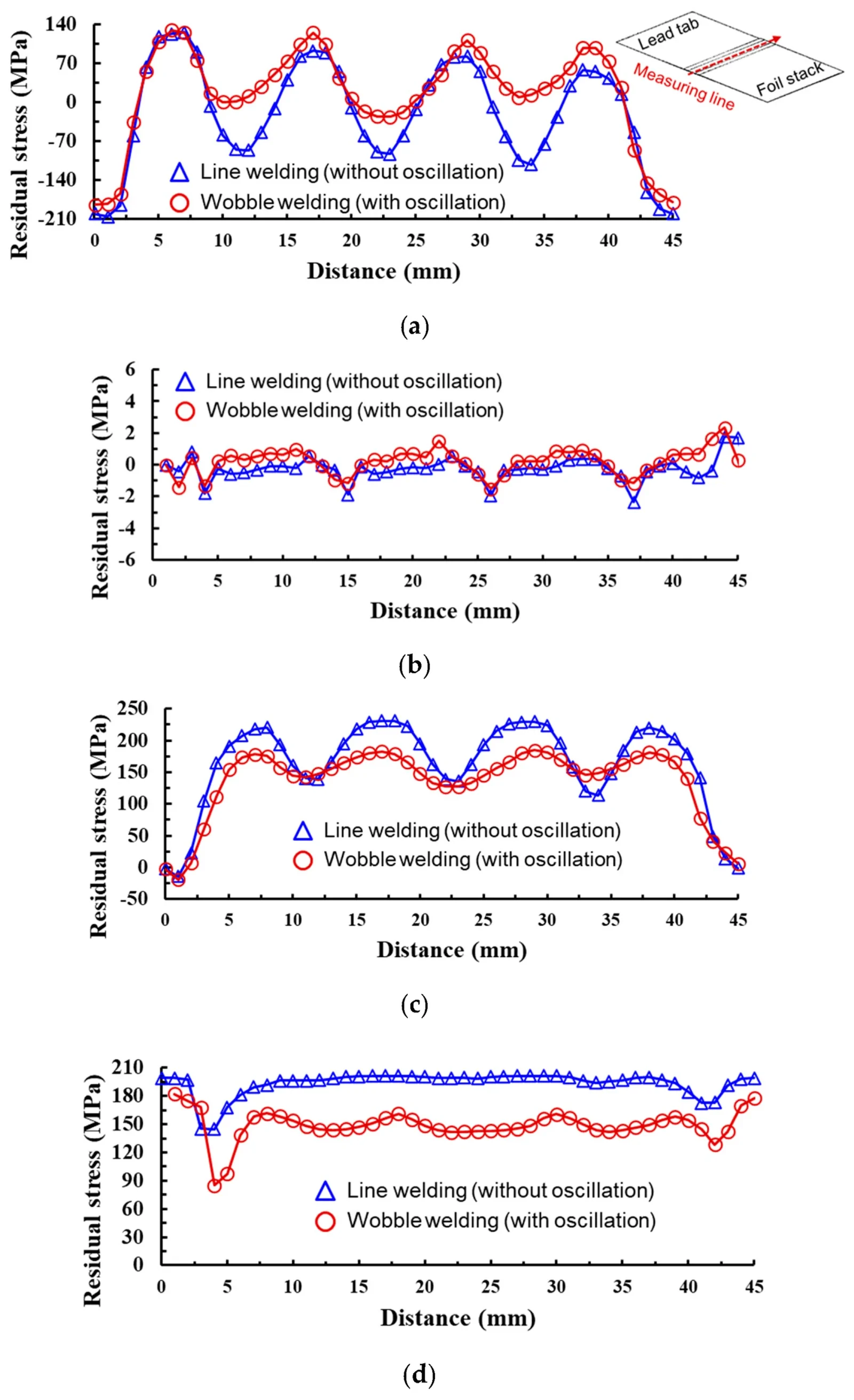
Comparison of residual stress profile between line- and wobble-welded joints. (**a**) σxx (width direction), (**b**) σyy (thickness direction), (**c**) σzz (longitudinal direction) and (**d**) von Mises equivalent stress.

Figure 11 shows the welding residual stress profiles along the longitudinal direction at the top surface of the weld line for line- and wobble-welded joints. As shown in Figure 11a, in the case of residual stress in the width direction, the line-welded joint exhibits an average tensile stress of 92 MPa at the peak value of the stitch seam, while compressive stress of around −100 MPa was observed in the pitch between the stitch seams. In contrast, the wobble-welded joint exhibits an average tensile stress of 114 MPa at the peak value of the stitch seam, which is about 24% higher compared with line welding. The pitch between the stitch seams has a compressive stress of around −12 MPa, which is reduced by approximately 88% compared with line welding. It can be inferred that the reduction in compressive stress in the material around the pitch between the stitch seams is due to the pre-heating and post-heating effects induced by the application of beam oscillation. Figure 11b shows the residual stress in the thickness direction; both line- and wobble-welded joints show the stress values close to 0 MPa, as previously observed in Figure 10b. For the residual stress in the longitudinal direction (Figure 11c), the line-welded joint exhibits an average tensile stress of 225 MPa at the peak value of the stitch seam. In contrast, the wobble-welded joint exhibits an average tensile stress of 181 MPa at the peak value of the stitch seam, which is reduced by approximately 20% compared with the line-welded joint. As shown in Figure 11d, the von Mises stresses for the line- and wobble-welded joints are 199 MPa and 141 MPa at the center of the weld line, respectively. In summary, it was found that the magnitude of the residual stresses follows the order of longitudinal > width > thickness direction, which indicates that the stress component in the longitudinal direction has a dominant influence on the welding residual stress. Specifically, the wobble-welded joint exhibited reduced welding residual stress due to a uniform thermal gradient along the longitudinal direction.

#### 3.2.2. Plastic Strain Characteristics

**Figure 12:**
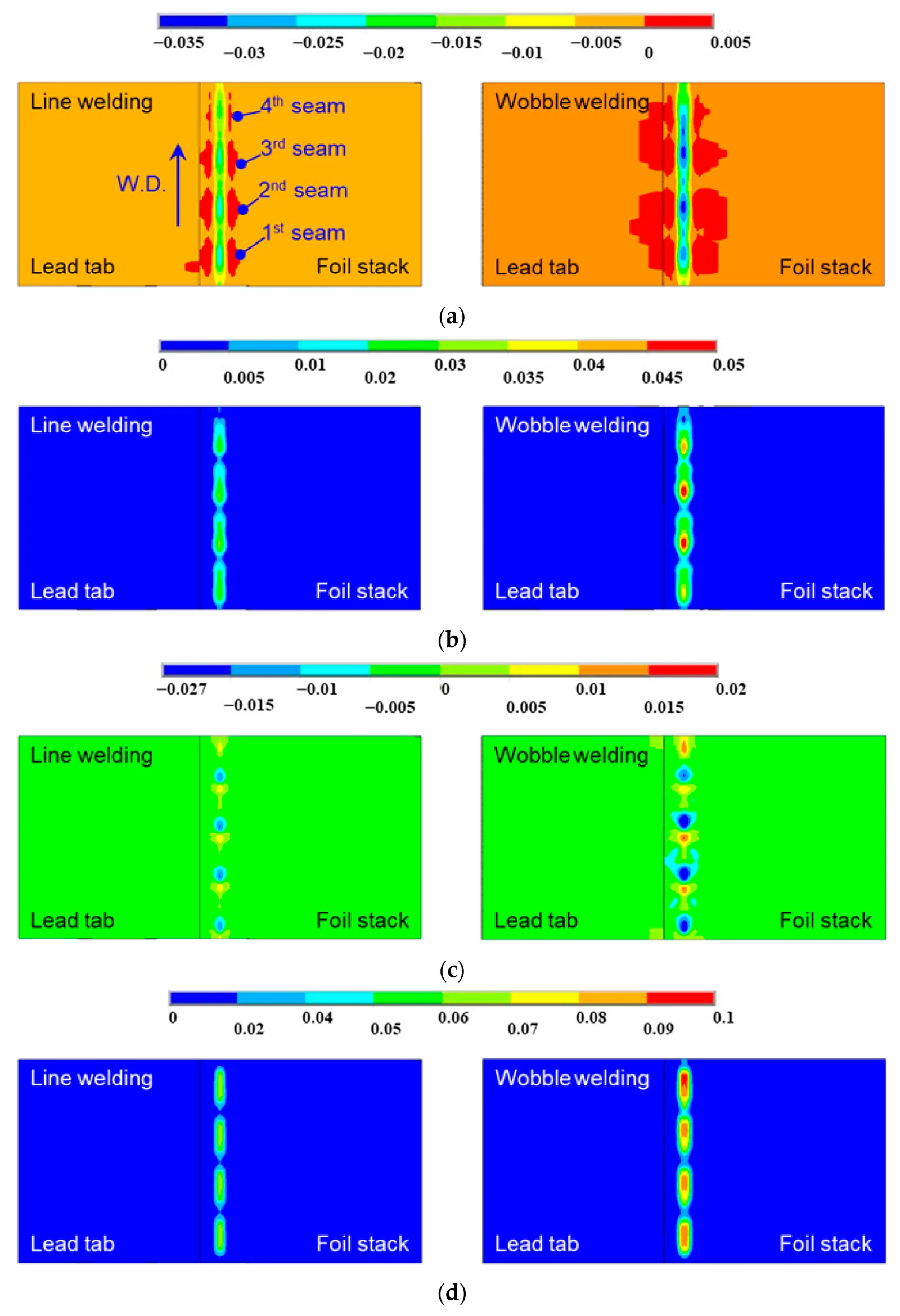
Comparison of plastic strain distributions between line- and wobble-welded joints. (**a**) εxx (width direction), (**b**) εyy (thickness direction), (**c**) εzz (longitudinal direction) and (**d**) equivalent plastic strain.

Figure 12 shows the comparison of plastic strain distribution for plastic strain components in different directions for line- and wobble-welded joints. In the case of plastic strain in the width direction (Figure 12a), the line- and wobble-welded joints exhibit plastic strains ranging from −0.028 to −0.017 and −0.032 to −0.028, respectively. This increase in compressive plastic strain can be attributed to the increased molten area due to beam oscillation, as previously observed in Figure 8. Unlike the typical plastic deformation behavior of welded joints, it can be observed from Figure 12b that the plastic strain in the thickness direction of the weld joint is in the tensile direction. It can be supposed that the plastic deformation occurs more freely in the length and width directions, while the thickness direction has limited space for contraction due to its ultra-thin thickness. As a result, the contraction in the length and width directions becomes dominant, leading to tensile plastic strain in the thickness direction as it is pulled by these contractions. The line- and wobble-welded joints exhibit plastic strains ranging from 0.029 to 0.033 and 0.035 to 0.050, respectively. The longitudinal component of plastic strain along the weld line exhibits both tensile and compressive plastic strain (Figure 12c). This result indicates that as the stitch seams contract during solidification, the material in the pitch area is physically pulled. This causes tensile plastic strain to occur in the pitch region as it is subjected to tension. The line- and wobble-welded joints exhibit plastic strains ranging from −0.016 to −0.013 and −0.025 to −0.016, respectively. As shown in Figure 12d, the equivalent plastic strain for the line and wobble joints exhibits plastic strains ranging from 0.065 to 0.068 and 0.082 to 0.094, respectively.

**Figure 13:**
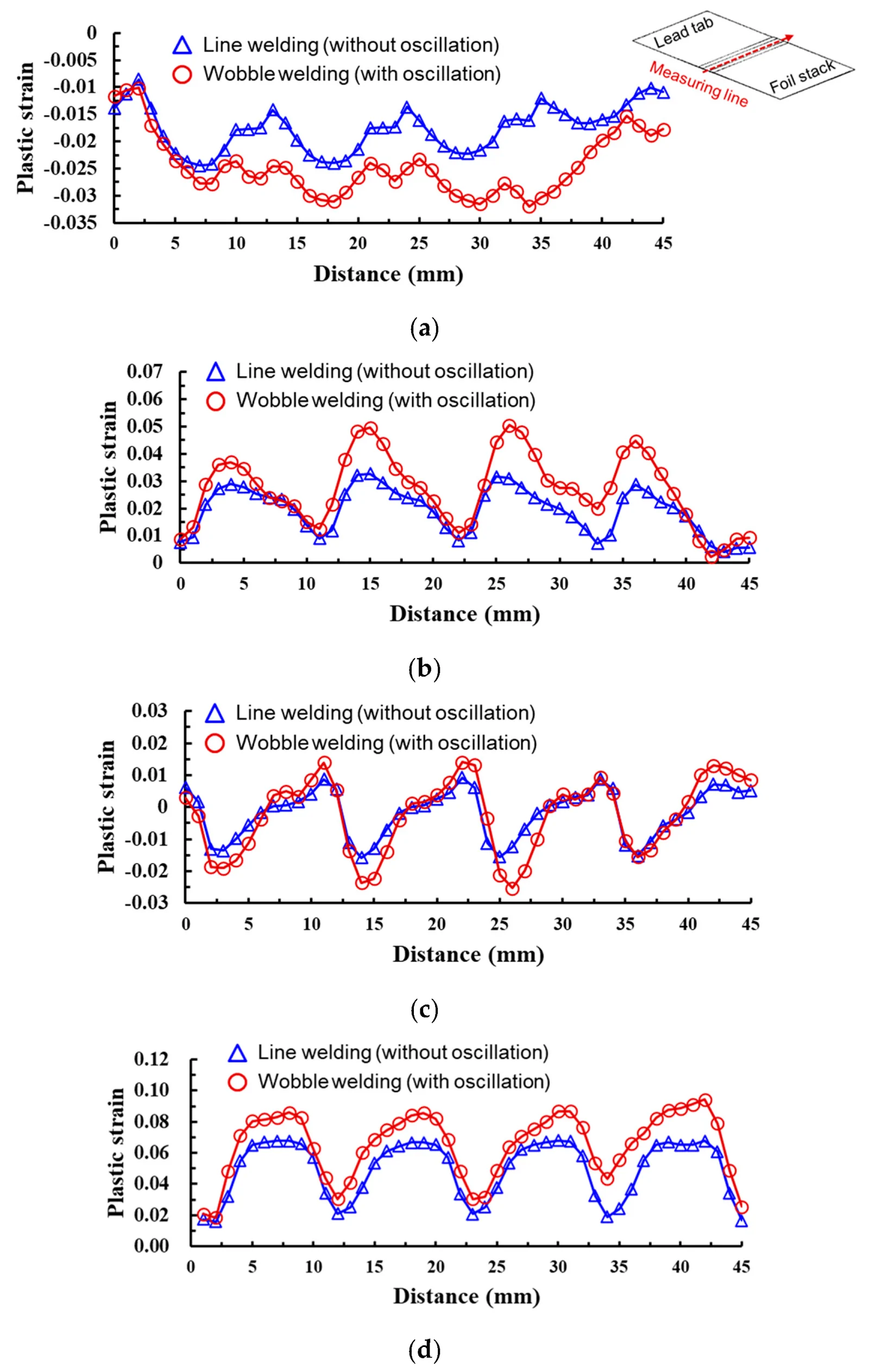
Comparison of plastic strain profile between line- and wobble-welded joints. (**a**) εxx (width direction), (**b**) εyy (thickness direction), (**c**) εzz (longitudinal direction) and (**d**) equivalent plastic strain.

Figure 13 shows the plastic strain distribution profiles along the longitudinal direction at the top surface of the weld line, including plastic strain components in different directions for line- and wobble-welded joints. For the plastic strain in the width direction for both line- and wobble-welded joints (Figure 13a), the line- and wobble-welded joints exhibit a maximum compressive plastic strain of −0.024 and −0.032, respectively. This is about 33% higher in wobble welding compared with line welding. Figure 13b shows the plastic strain in the thickness direction for both line- and wobble-welded joints. The line- and wobble-welded joints exhibit maximum tensile plastic strains of 0.033 and 0.051, respectively. As shown in Figure 13c, for the plastic strain in the longitudinal direction for both line- and wobble-welded joints, the line- and wobble-welded joints exhibit maximum compressive plastic strains of −0.016 and −0.025, respectively. This is about 56% higher in wobble welding compared with line welding, whereas the maximum tensile plastic strain in the pitch area between the stitch seams is 0.009 for the line-welded joint and 0.014 for the wobble-welded joint, which is about 56% higher in wobble welding compared with line welding. These results indicate that the relatively larger molten pool formed by beam oscillation causes higher tension during the contraction of material. Figure 13d presents the maximum equivalent plastic strain for the line- and wobble-welded joints at 0.068 and 0.094, respectively. This is about 38% higher in wobble welding compared with line welding. From the results, it was found that the magnitude of the plastic strain follows the order of thickness > width > longitudinal direction. However, in the case of the thickness direction, there are restrictions on contraction due to its thin thickness, and the plastic strain can be attributed to the contraction in the length and width directions. Therefore, it can be assumed that the plastic strain in the thickness direction is not considered a significant factor.

## 4. Conclusions

In this study, a three-dimensional sequentially coupled thermo-elastic-plastic finite element analysis was successfully developed to investigate the thermal behavior, residual stress evolution, and plastic strain distribution in laser-welded joints of 60-layer Cu foil stacks joined to a 0.3 mm Cu lead tab. The comparative effects of conventional line welding and wobble beam oscillation welding were quantitatively evaluated. The results can be summarized as follows:

1. The thermal conduction analysis results revealed that the peak temperatures of the wobble- and line-welded joints were 2577 K and 2060 K, respectively. Furthermore, a gradual thermal gradient was achieved along the width, length, and thickness directions of the wobble-welded joint due to the enhanced melt pool flow and uniform heat distribution.
2. The components of welding residual stress are in the following order: longitudinal (σ_ZZ_) > width (σxx) > thickness (σyy) direction. Moreover, the von Mises equivalent stress in the wobble-welded joint was observed to be 141 MPa, which is about 29% lower compared with the line-welded joint.
3. In conclusion, laser beam oscillation redistributes thermal energy and promotes uniform thermal cycles, effectively suppressing localized residual stress concentration in multilayer foil-to-tab joints. These numerical insights provide a fundamental basis and process design guidelines for optimizing beam oscillation strategies to enhance the structural reliability and fatigue performance of multilayer current collector joints in lithium-ion battery manufacturing. However, these findings are limited to the simulated welding parameters and cannot be directly generalized to the entire wobble-welding parameter window.
4. Under the investigated welding conditions, wobble welding exhibited a lower predicted residual stress than line welding, which may provide a potential advantage in the mechanical reliability of battery tab joints. In pouch-type battery cells, welded tab joints may experience additional mechanical loading due to external impact and cell swelling. Under such conditions, the lower residual stress associated with wobble welding may be beneficial by reducing the initial stress state of the welded joint before external loading is applied. However, wobble welding also exhibited higher plastic strains, indicating greater accumulated permanent deformation during welding. Such localized plastic deformation may increase the susceptibility of the welded joint to micro-crack initiation and deterioration of mechanical durability under subsequent impact or swelling-induced loading.

## Figures and Tables

**Table 1:** Parametric cases with and without beam oscillation.

Subsystem/Unit	Parameter	Specification/Value
Case 1	Laser power	2000 W
	Welding speed	150 mm/s
	Focal depth	0 mm (just focused on surface)
Case 2	Laser power	2000 W
	Welding speed	50 mm/s
	Focal depth	0 mm (just focused on surface)
	Wobble amplitude	0.8 mm
	Oscillation frequency	313 Hz
	Overlap	80%

## Data Availability

The original contributions presented in this study are included in the article. Further inquiries can be directed to the corresponding author.
